# Case Report: PROS1 (p.Leu584Arg) pathogenic mutation causes portal and superior mesenteric venous thromboembolism

**DOI:** 10.3389/fcvm.2023.1277676

**Published:** 2023-11-14

**Authors:** Peng Ding, Yuan Zhou, Kai-Chen Zhang, Sheng Li, Kun-lan Long, Jun Chen, Ying-jie Chen, Pei-yang Gao

**Affiliations:** ^1^ Department of Critical Care Medicine, Hospital of Chengdu University of Traditional Chinese Medicine, Chengdu, China; ^2^ Department of Geriatric Medicine, General Hospital of Western Theater Command, PLA, Chengdu, China

**Keywords:** portal vein thrombosis, superior mesenteric vein thrombosis, PROS1, protein S deficiency, case report, pathogenic mutation

## Abstract

**Background:**

Genetic and acquired risk factors are fundamental to developing venous thromboembolism. Autosomal dominant protein S deficiency caused by pathogenic mutations in the PROS1 gene is a well-known risk factor for thrombophilia.

**Case presentation:**

We report a 30-year-old male patient who presented to the hospital with portal vein thrombosis. The patient had a history of abdominal pain for one month. Abdominal vascular CT showed venous thrombosis in the portal vein and superior mesenteric vein. He was diagnosed with “portal and superior mesenteric vein thrombosis, small bowel obstruction and necrosis, acute upper gastrointestinal bleeding (UGIB), hemorrhagic shock.” Serum protein S levels were decreased, and gene sequencing revealed a heterozygous missense mutation in PROS1, c.1571T > G (p.Leu584Arg). The patient received anticoagulation therapy with Enoxaparin Sodium and rivaroxaban, transjugular intrahepatic portosystemic shunt (TIPS), and ICU treatments. Although the patient had a severe bleeding event during anticoagulation therapy, he recovered well after active treatment and dynamic monitoring of anti-Xa.

**Conclusion:**

Hereditary protein S deficiency caused by a mutation in the PROS1 gene is the genetic basis of this patient, and Enoxaparin Sodium and rivaroxaban have been shown to be highly effective.

## Introduction

Venous thromboembolism (VTE) includes deep venous thrombosis (DVT) and pulmonary embolism (PE) in the lower extremities or pelvis, which can be life-threatening in severe cases. VTE in critically ill patients can increase the duration of mechanical ventilation and hospitalization in the intensive care unit (ICU) (1) and is associated with poor prognosis. Acquired or genetic risk factors can usually cause VTE. Acquired risk factors associated with VTE in critically ill patients include advanced age, obesity, active malignancy, history of VTE, and recent history of surgery. Sepsis, failure to use anticoagulant drugs for venous thromboprophylaxis, central venous catheterization, invasive mechanical ventilation, and use of vasoactive medications are specific factors for increased risk of VTE in critically ill patients (2). Genetic risk factors can predispose patients to thrombophilia, including factor V Leiden and prothrombin G20210A mutations, as well as deficiency of antithrombin, protein C, and protein S (3). Protein S deficiency is associated with venous thrombosis, and pathogenic mutations in the PROS1 gene can lead to autosomal dominant thrombophilia due to protein S deficiency (4). A missense mutation in the coding region of the PROS1 gene caused by c. 1751T > G (p.Leu584Arg) has not been reported in the literature or the large-scale population frequency database gnomAD. Here, we present a young man diagnosed with severe portal and superior mesenteric venous thromboembolism. Genetic sequencing revealed a missense mutation in PROS1 (p.Leu584Arg).

## Case presentation

**Figure 1:**
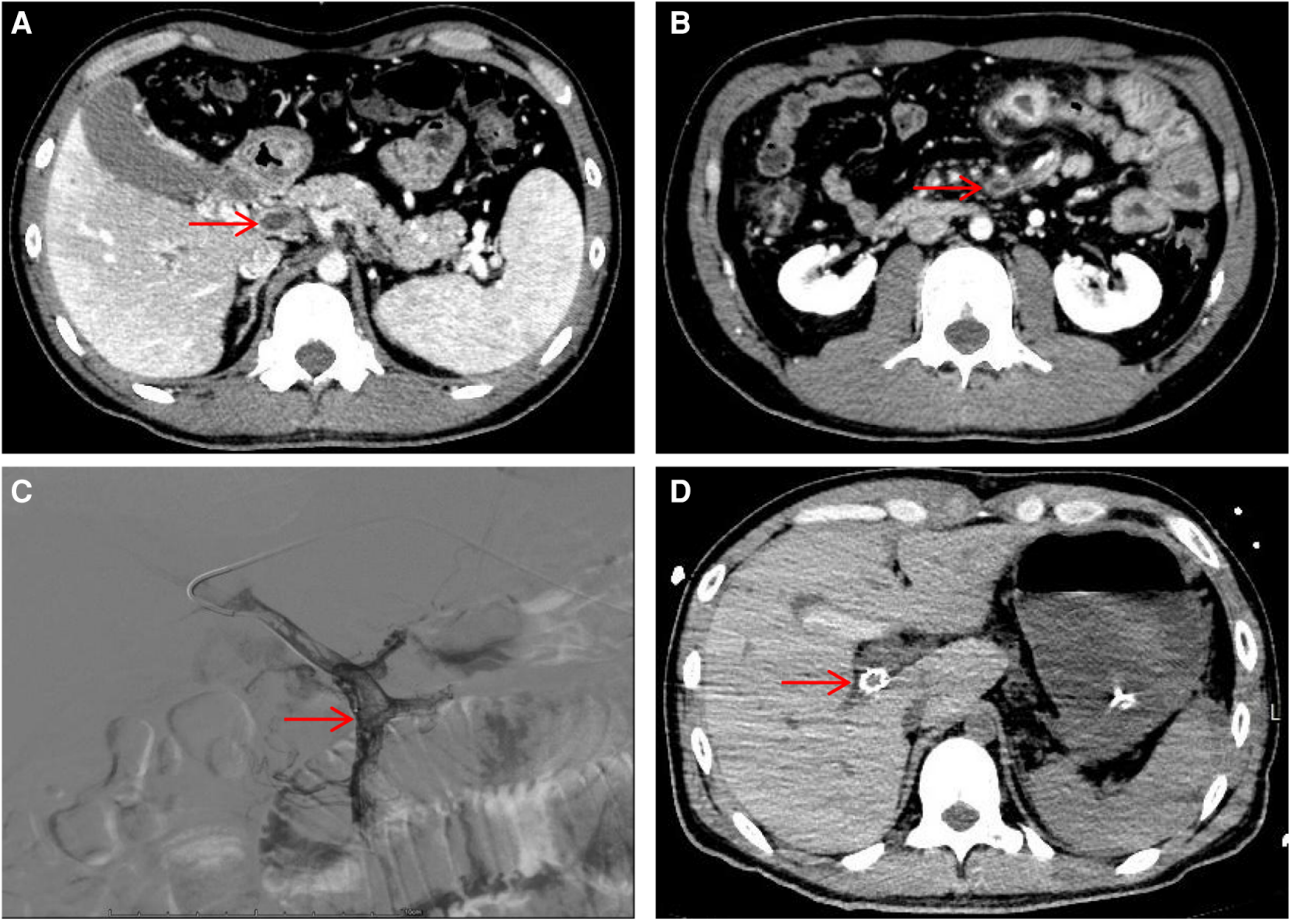
Hepatic portal vein CT angiography and digital subtraction-angiography (DSA). (**A**) Showing portal vein thrombosis (red arrow). (**B**) Showing superior mesenteric vein thrombosis (red arrow). (**C**) DSA showed filling defects in the portal and superior mesenteric veins. (**D**) Visualization of stent shadow after TIPS.

A 30-year-old male patient was admitted to the Interventional Oncology Department of our hospital on February 24, 2023, due to “repeated abdominal pain for one month, aggravated for 2 h”. One month before admission the patient presented with unexplained abdominal pain and visited Lhasa People's Hospital. Abdominal enhanced CT showed thrombosis of the portal vein, splenic vein, and superior mesenteric vein, with ischemic changes in the small intestine and localized peritonitis. The patient was treated with oral rivaroxaban. Day 1 of admission, the patient visited the emergency department of our hospital due to severe and unbearable abdominal pain. Emergency ultrasound showed thrombosis of the portal vein system. Abdominal vascular enhanced CT results showed thrombosis of the central portal vein, the left and right branches of the portal vein, and the superior mesenteric vein (Figures 1A,B), and portal hypertension with collateral circulation formation. The patient was healthy before this onset and denied a family history of venous thrombosis. The patient had a history of smoking.

**Figure 2:**
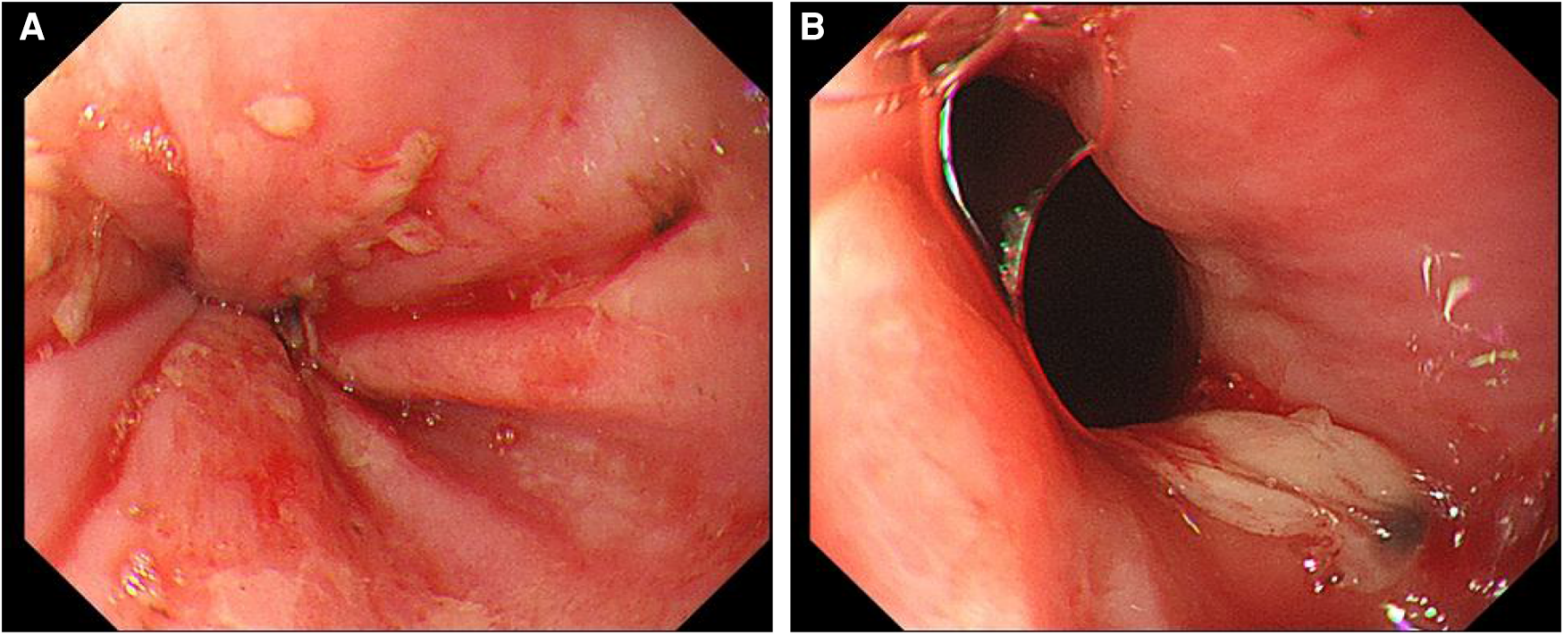
Upper gastrointestinal endoscopy. (**A**) Scattered erosive focus in the lower esophagus, with a small amount of oozing blood and visible venous exposure. (**B**) A large ulcer in the lower esophagus near the pyloric ostium.

After admission, the patient's blood tests showed that hemoglobin was 177 g/L (normal range 130–175), hematocrit was 50.6% (normal range 40–50), platelets were 200 × 10^9^/L (normal range 100–300), total bilirubin was 34.5 umol/L (normal range 0–26). Direct bilirubin 13.4 umol/L (normal range 0–8), indirect bilirubin 21.1 umol/L (normal range 0–17), creatinine 95 mmol/L (normal range 57–97), prothrombin time 19.1 s (normal range 11–14.3), Activated partial thromboplastin time 55.9 s (normal range 31.5–43.5), plasma fibrinogen 5.1 g/L (normal range 2.0–4.0), D-dimer 1.47 ug/ml (normal range 0–0.5). The patient was given low molecular weight heparin (LMWH) (i.e., enoxaparin sodium, CLEXANE), 40 mg every 12 h. Day 24 of admission, the patient vomited dark red bloody liquid about 500ml. Portal hypertension and rupture and bleeding of esophageal and gastric varices were considered, and the patient received variceal embolization and TIPS (Figures 1C,D). Day 27 of admission, the patient developed shock, and the hemoglobin decreased to 38 g/L (normal range: 130–175, 177 g/L on admission). Gastroscopy showed erosion, bleeding, and varices in the lower esophagus (Figures 2A,B). The patient was diagnosed with acute upper gastrointestinal bleeding and hemorrhagic shock and transferred to our hospital's ICU.

**Figure 3:**
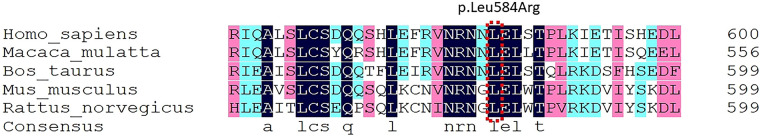
Species conservation analysis using DNAMAN (Lynnon Biosoft, Quebec, Canada) software showed that the mutation sites of PROS1 gene p.Leu584Arg were homo sapiens, Macaca mulatta, Bos Taurus, Mus musculus, and Species such as rattus norvegicus are highly conservative in evolution.

After being transferred to the ICU the patient was immediately treated with invasive ventilator-assisted respiration, fluid resuscitation, blood transfusion, and maintenance of blood pressure with norepinephrine. Day 33 of admission, after the bleeding became stable, the patient was given anticoagulation with Enoxaparin Sodium Injection (Table 1). Day 46 of admission, the patient developed shock again, and abdominal CT showed a large heterogeneous density mass in the anterior part of the spleen, with a more significant level of about 6.0 cm × 5.5 cm, mildly heterogeneous enhancement on enhanced scan; patchy iso-slightly high-density shadows in the left paracolic sulcus, with possible hematoma (Figure 3). Considering splenic rupture and hemorrhagic shock, the general surgeon performed a splenectomy immediately, during which the small intestine about 80cm from the ligament of Treitz was resected (local scarring and contracture of the mesenteric and intestinal cavity were observed during the operation, resulting in complete obstruction of the upper intestinal cavity, and some small intestine necrosis). Day 48 of admission, the patient was stable with no active bleeding and was given subcutaneous anticoagulation therapy with enoxaparin sodium injection again, while the concentration of Anti-Xa was monitored (the therapeutic dose range of subcutaneous injection was 0.5–1.0I U/ml) (Table 1). Protein S measurement was performed: 22% (normal range: 77–143), and lupus anticoagulant, plasminogen, antithrombin III, and protein C measurements were all within the normal range.

**Table 1:** Use of enoxaparin sodium injection after admission to the ICU.

Date	Enoxaparin Sodium Injection		Anti-Xa Testing (IU/ml)
	Dose (mg)	Interval	
March 29–April 4	40	Q12 h	Not tested
April 5–10	60	Q12 h	Not tested
April 13–15	40	Q12 h	0.27
April 16–26	70	Q12 h	1.23

### Follow-up

Day 61 of admission, the patient's condition improved and he was transferred to the acupuncture department for rehabilitation treatment. Following arrival at the acupuncture department, the patient was treated with rivaroxaban (20 mg by mouth once daily). After 7 months of admission, the patient remained stable and clinically improved, with no further bleeding or thrombosis.

### Results of gene detection and pathogenicity analysis of mutation site

The case of gene sequencing using sequence capture high-throughput sequencing technologies, hereditary blood clots on the human genome and other relevant genes coding regions, coagulopathy sequencing. The average sequencing depth of the obtained next-generation sequencing data on the exons, upstream and downstream 5bp sequences of the nuclear genes included in this study was 90X or greater, and the proportion of sequences with sequencing depth greater than 20X was about 98%. Genetic testing for thrombophilia revealed a heterozygous mutation at base 1,751 of the PROS1 gene on chromosome 3 short arm, a substitution of a thymidine deoxyribonucleotide for a guanine deoxyribonucleotide, resulting in a substitution of arginine for leucine at amino acid 584 of the mRNA (c.1751T > G; p.Leu584Arg). Other genes associated with thrombosis include: Jak-2 (myeloproliferative neoplasm), F2 (prothrombin G20210A), F5 (factor V Leiden), SERPINC1 (antithrombin Ⅲ) and PROC(protein C) were all negative. In silico bioinformatics tools (SIFT (5), Polyphen2 (6), FATHMM (7), Mutation assessor (8)) predicted that PROS1 gene c.1751T > G (p.Leu584Arg) was a pathogenic mutation (Table 2). The species conservation analysis of the human PROS1 gene p.Leu584Arg locus showed that it was highly conserved in the evolution of different species (Figure 3).

**Table 2:** PROS1 gene c.1,751T > G (p.Leu584Arg) was predicted by SIFT, Polyphen2, FATHMM and Mutation assessor software.

Gene mutation site	Software scoring and predicted							
	SIFT	predicted	Polyphen2	predicted	FATHMM	predicted	Mutation assessor	predicted
c.1,751T > G	0.001	Pathogenic	1	Pathogenic	0.9103	Pathogenic	2.94	Pathogenic

## Discussion and literature review

Here, we report a patient with portal and superior mesenteric venous thromboembolism. Genetic testing revealed that there was a heterozygous mutation at base 1,751 of the PROS1 gene on the short arm of chromosome 3, resulting in the substitution of amino acid 584 of its mRNA from arginine to leucine. He accepted vascular intervention and anticoagulation. After treatment with rivaroxaban Enoxaparin Sodium Injection, no adverse events (e.g., bleeding and re-embolization) occurred during the 6-month follow-up period.

All venous thrombosis is multifactorial and caused by the components of Virchow's triad: venous stasis, vascular injury, and hypercoagulability. The portal vein system's low pressure, slow flow rate, and high hemodynamics result in a unique vascular environment that creates favorable conditions for thrombosis. Most of the portal vein thrombosis is secondary to cirrhosis, while non-cirrhotic portal vein thrombosis is less common but still produces an adverse severe prognosis. Hypercoagulability is considered the primary pathophysiological mechanism of both types of portal vein thrombosis. The most common potential hypercoagulable state-related risk factors for non-cirrhotic portal vein thrombosis are myeloproliferative neoplasm, G20210A mutation in the prothrombin gene, and antiphospholipid syndrome (9). However, none of the above risk factors were confirmed in the patient reported in this study. Genetic testing revealed that the patient had a mutation in the PROS1 gene on the short arm of chromosome 3, resulting in protein S deficiency, which was the genetic risk factor that ultimately led to the occurrence of portal vein thrombosis. Intestinal infarction is the most direct severe complication of portal-mesenteric vein thrombosis, with a mortality rate as high as 60% (10). Unfortunately, the patient developed intestinal obstruction and necrosis and was treated with surgical resection. However, after a long rehabilitation period, the patient's bowel function recovered, and he could take a liquid diet through the mouth independently.

Thrombophilia is a genetic disorder that increases the risk of VTE. Portal vein and/or mesenteric vein thrombosis are rare in young patients and are more difficult to treat than typical DVT or pulmonary embolism (11). Protein S is a vitamin K-dependent protein encoded by the human PROS1 gene, and PROS1 heterozygous deficiency is associated with an increased risk of thrombosis (12). Protein S has anticoagulant effects: inhibiting the prothrombin complex (factor Xa, factor Va, and phospholipids) that convert prothrombin to thrombin, and the tenase complex (factor IXa, factor VIIIa, and phospholipids) that convert factor X to factor Xa (13). Studies from China have shown that 8.5% (51/603) of the Han population were detected to be deficient in free protein S antigen, 30 of them were identified with pathogenic mutations by direct sequencing, and the LGR2 domain is the hotspot mutation region of the protein (14). The PROS1 gene is located on the short arm of chromosome 3, which contains 15 exons spanning more than 100,000 base pairs, and its pathogenic mutations are considered to cause hereditary thrombophilia. Results of a molecular basis study on protein S deficiency in China showed that c. 1095T > G (p.Asn365Lys) and c. 1229C > A (p.Pro410His) mutations affect the structural integrity of protein S (15). The c. 1571T > G (p.Leu584Arg) mutation in this patient has not been reported in the current literature. We believe that the amino acid sequence of protein S has been changed due to this nucleotide sequence mutation, and this has an adverse effect on the structure and function of protein S. It further affects the interaction between protein S and other coagulation factors, and as there is an abnormal coagulation process this leads to thrombosis.

The EASL guidelines recommend that all patients with visceral thrombosis should be anticoagulant indefinitely, with unfractionated heparin and LMWH preferred (10). Given the parenteral route of administration of LMWH, patient adherence and quality of life can be an issue, so vitamin K antagonists (VKA) can be used for long-term treatment (16). Compared with VKA, direct oral anticoagulants (DOAC) have shown similar efficacy and better safety in patients with acute venous thrombosis at routine sites and are currently recommended as first-line treatment. The risk-benefit of DOAC could be similarly favorable in patients with splanchnic vein thrombosis (17). DOAC is still widely used in the prevention and treatment of various types of VTE, mainly including factor Xa inhibitors (rivaroxaban, apixaban, and edoxaban) and direct thrombin inhibitors such as dabigatran. Meta-analysis showed the overall safety and effectiveness of DOAC in patients with VTE and thrombophilia (18). The patient developed portal and superior mesenteric vein thrombosis, esophageal and gastric variceal bleeding, and splenic rupture, all of which occurred during anticoagulant therapy. This made our anticoagulation therapy more difficult to control, requiring repeated consideration between thrombosis recurrence and increased risk of bleeding. In this case, DOAC was not applicable, and treatment decisions mainly depended on the presence of predisposing factors rather than the absolute risk of bleeding. Although bleeding may have a severe negative impact on the patient's quality of life and even lead to death, if the risk factors for venous thrombosis persist, even though the patient has a high risk of bleeding, the benefits of prolonged anticoagulation therapy still outweigh the risk of bleeding. Therefore, LMWH sodium was used for anticoagulation therapy (19). After repeated monitoring of FXa levels, the LMWH sodium dosage was adjusted to reach a safe and effective anticoagulation.

## Limitations

Patient had significantly lower levels of protein S when first detected and were not reviewed after two weeks. Although the patient had liver disease and acute thrombosis, which would affect the decrease of protein S level, the test results of protein C, antithrombin III, and lupus anticoagulant detected at the same time as protein S were all within the normal range. Therefore, we believe that the decrease in protein S in this patient is the result of his gene mutation. In addition, this case is based on the specific conditions and circumstances of the patient's visit and may not fully reflect the situation of the entire patient population and so needs to be interpreted with caution.

## Conclusion

Hereditary protein S deficiency caused by PROS1 gene mutation is the genetic basis of this patient. LMWH and rivaroxaban have good efficacy for this disease. Taking targeted diagnostic and therapeutic measures is significant for the patient's clinical outcome.

## Data Availability

The original contributions presented in the study are included in the article/Supplementary Material, further inquiries can be directed to the corresponding author.
